# The Effect of Home Bleaching Gel with Chitosan on Tooth Color and Mineral Alteration

**DOI:** 10.3390/gels11110933

**Published:** 2025-11-20

**Authors:** Görkem Kervancıoğlu, Derya Gürsel Sürmelioğlu

**Affiliations:** 1 Şehitkamil Oral and Dental Health Center, Gaziantep 27000, Turkey; 2 Department of Restorative Dentistry, Faculty of Dentistry, Gaziantep University, Gaziantep 27310, Turkey

**Keywords:** theobromine, chitosan, carbamide peroxide, hydrogen peroxide, color change, SEM-EDX

## Abstract

This study aimed to compare tooth mineralization and color changes achieved with two experimental bleaching gels containing chitosan and theobromine (16% CP or 6% HP, Group 1, 2) with the FGM Whiteness Perfect (16% CP) (Group 3) and BioWhitenProHome (6% HP) (Group 4). Ninety-six maxillary central teeth were divided into two groups for color and mineral evaluations. These groups were then further divided into four subgroups according to the bleaching agent (n = 12). Mineral analysis was performed with SEM-EDX before the bleaching, at the end of the treatment, and two weeks after treatment ended to assess changes. Color measurement was performed with a spectrophotometer before bleaching, on the 7th day of treatment, 24 h after final treatment, and two weeks after treatment ended. No significant difference among the groups was found in color change (*p* > 0.05), while mineralization differed significantly (*p* < 0.05). The ΔE_003_ values of Group 4 and Group 2 were found to be close to each other. The highest calcium loss was detected in Group 3, whereas the most pronounced decrease in phosphorus values was observed in Group 4. Using theobromine and chitosan can provide clinicians with positive results for bleaching treatments, such as using lower HP concentrations and avoiding side effects.

## 1. Introduction

Many studies have compared different tooth-bleaching procedures in terms of treatment effectiveness, continuity of effectiveness, tooth sensitivity, and general patient satisfaction by evaluating numerous criteria, such as the application method, the agent used, the agent concentration, and the treatment duration. Agents containing hydrogen peroxide (HP) and carbamide peroxide (CP) are preferred in bleaching applications. Pereira et al. investigated the effects of office and home bleaching applications on tooth color changes and reported greater color relapse after 6 months with home applications [1]. Another study assessed office (38% HP) and home (10% CP) bleaching agents in terms of the agent, method, sensitivity, and patient satisfaction and revealed greater tooth color changes, reduced sensitivity, and better patient satisfaction with home applications [2]. A third study assessed the effectiveness of home (10% and 20% CP) and office (35% and 38% HP) bleaching agents, as well as the associated tooth sensitivity, and revealed that sensitivity was highest after applying the 20% CP treatment and lowest after the office bleaching treatments, with no difference in efficacy or the final color results [3]. The literature reveals discrepancies in effectiveness, sensitivity, and patient satisfaction regarding office and home bleaching methods, with each method having its advantages and disadvantages [1,2,4].

HP produces free radicals that can damage tissues due to their high oxidizing activity [5]. Many studies have revealed the undesirable effects of HP after bleaching applications, including changes in tooth structure [6], pulpal tissues [7], and oral mucosa [8]. Cytotoxic effects have been observed after bleaching with agents containing high HP levels; for example, in a study comparing low and high HP concentrations in bleaching applications, Lilaj et al. [9] emphasized that cytotoxic effects increase as the HP concentration increases. The 2011/84/EU European Directive indicates that a maximum HP concentration of 6% is safe for tooth-bleaching agents [10]. Due to the side effects of numerous HP applications and in line with this directive, we developed a bleaching gel containing a low HP concentration.

Chitosan, one of the most abundant biopolymers in the world, is natural, biocompatible, and non-toxic [11]. Chitosan contributes to oral tissue wound healing [12] and reduces enamel demineralization, caries formation, and acid-induced erosion [13], along with preventing dental plaque formation [14] and exerting antimicrobial effects on oral pathogens [15]. Thus, chitosan is extensively applied in dentistry [16]. We added chitosan to our experimental gels as a carrier and thickening agent, obtaining additional benefits from its remineralizing effects and bioadhesive properties [17,18].

Theobromine is an alkaloid found in chocolate and cocoa [19]. Because it prevents demineralization and possesses remineralizing effects, theobromine is thought to contribute to the remineralization of dental hard tissues [20]. Theobromine and fluorine have been shown to contribute to remineralization, achieving similar levels of apatite formation and enamel surface hardening [21]. Thus, numerous studies have been conducted to investigate theobromine’s ability to prevent caries, and it was found to reduce Streptococcus mutans [22]. Given the above advantages, theobromine is an attractive enamel remineralization material. Therefore, theobromine was added to our experimental gels for its remineralizing and antikaryostatic effects [23,24].

Thus, aiming to reduce the side effects of bleaching treatments while obtaining effective bleaching results, we have developed an experimental home bleaching gel containing chitosan and theobromine. To ensure standardization, we selected gels containing 6% HP, 16% CP, or an equivalent concentration of the active agent (HP) for comparison with the new home bleaching gels.

In this study, the newly developed experimental bleaching gels were compared with the BioWhiten ProHome (6% HP) and FGM Whiteness Perfect (16% CP) home bleaching systems, and their effects on enamel mineral and tooth color changes were evaluated. Mineral changes were evaluated with a scanning electron microscope–energy dispersive X-ray (SEM-EDX) device, and color changes were evaluated with a spectrophotometry device. In addition, cone-beam computed tomography (CBCT) was used to determine the enamel thickness of all samples for standardization.

The null hypotheses were as follows:

(i) Adding theobromine to bleaching gels does not adversely affect tooth color changes or mineralization.
(ii) Adding chitosan to bleaching gels does not adversely affect tooth color changes or mineralization.

## 2. Results and Discussion

### 2.1. Analysis of Color Change Values

Color change values were calculated using the CIEDE2000 formula.

ΔE_001:_ Color change values from pre-application to 7 days after the start of application.

ΔE_002:_ Color change values from pre-application to 15 days after the start of application

ΔE_003:_ Color change values from pre-application to 14 days after the end of application (end of 28th day)

The comparisons of ΔE_00_ color change values within and between groups are shown in Table 1.

No significant differences were observed in the ΔE_001_, ΔE_002,_ and ΔE_003_ values in the between-groups comparison (*p* > 0.05).

In the group comparison, while a significant increase was observed in the ΔE_001_, ΔE_002_, and ΔE_003_ values across groups (*p* < 0.05), there was no significant difference between the ΔE_002_ and ΔE_003_ values (*p* > 0.05).

### 2.2. Analysis of Mineral Changes

Statistical analyses were performed by specifying the mineral content values of all groups as Ca_1_, P_1_ (SEM-EDX analyses performed before the bleaching applications), Ca_2_, P_2_ (after the bleaching applications), and Ca_3_, P_3_ (14 days after the end of the bleaching applications).

#### 2.2.1. Calcium Mineral Findings

The mean values (±sd) of Ca in all groups are shown in Table 2.

No significant difference was observed in Ca_1_ values in the between-groups comparison (*p* > 0.05). However, Ca_2_ and Ca_3_ values differed significantly among Groups 1–4 (*p* < 0.05).

In the group comparison, no significant difference in any Ca values was observed between Groups 1 and 2 (*p* > 0.05). However, a significant difference was observed between the Ca_1_ value and the Ca_2_ and Ca_3_ values in Groups 3 and 4 (*p* < 0.05).

The Ca values were increased in Groups 1 and 2 after bleaching but decreased in Groups 3 and 4.

#### 2.2.2. Phosphorus Mineral Findings

The mean values (±sd) of P in all groups are shown in Table 3.

No significant differences in the P_1_, P_2,_ and P_3_ values were found in the between-groups comparison (*p* > 0.05).

In the group comparison, no significant differences in P1, P2, P3 values were observed in Groups 1 and 2 (*p* > 0.05); however, a significant difference was found between the P1 value and the P2 and P3 values in Groups 3 and 4 (*p* < 0.05).

### 2.3. Evaluation of the SEM Images

**Figure 1:**
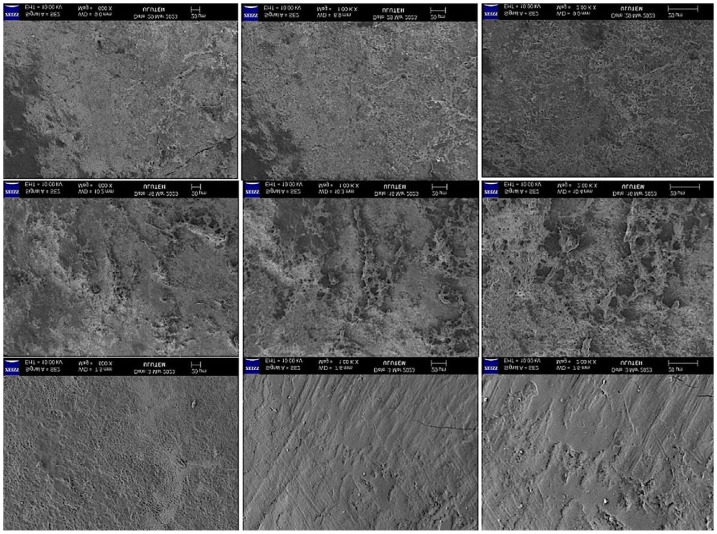
SEM image (×600 ×1000 ×2000) of the enamel surface of the experimental CP Gel Bleaching samples.

**Figure 2:**
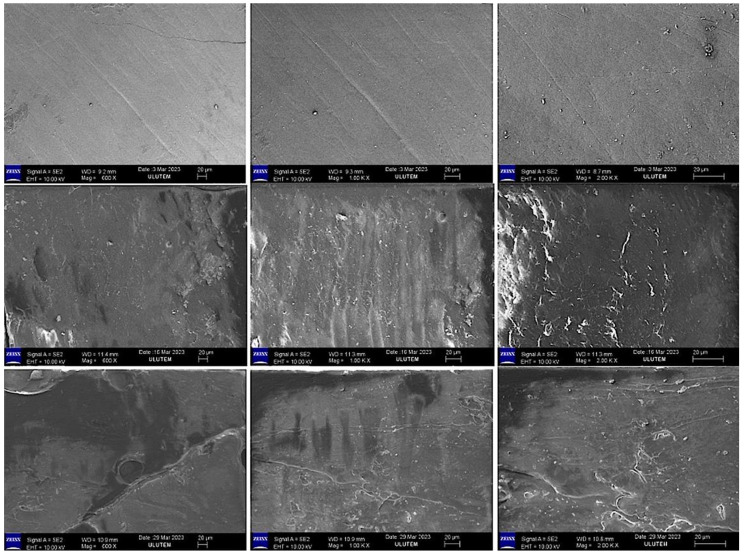
SEM image (×600 ×1000 ×2000) of the enamel surface of the experimental HP Gel Bleaching samples.

**Figure 3:**
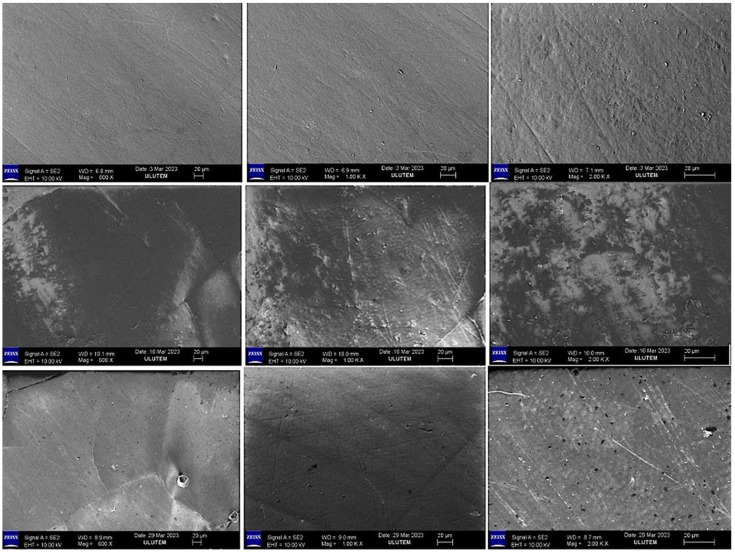
SEM image (×600 ×1000 ×2000) of the enamel surface of samples treated with BioWhiten ProHome Gel.

**Figure 4:**
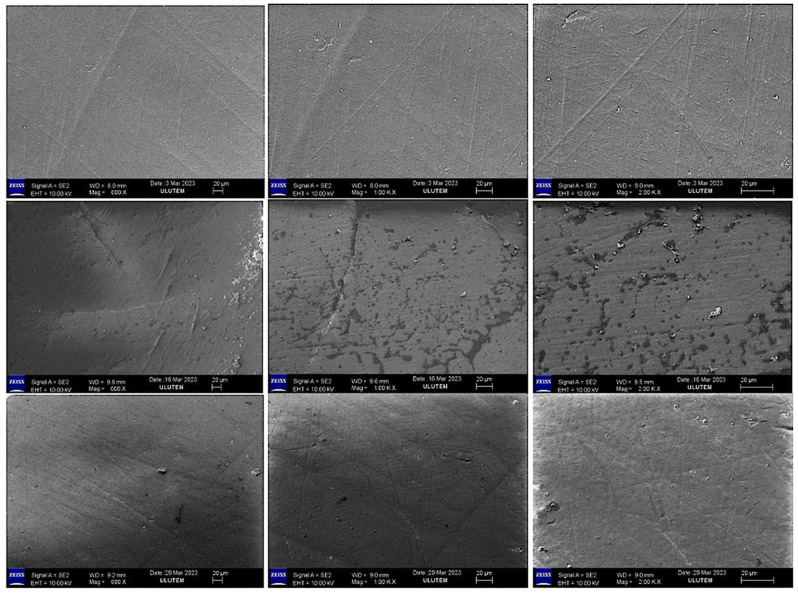
SEM image (×600 ×1000 ×2000) of the enamel surface of the samples treated with FGM Whiteness Perfect Gel.

SEM images (×600 ×1000 ×2000) of the mineral surfaces of bleached samples in all groups are shown in Figure 1, Figure 2, Figure 3 and Figure 4.

Bleaching agents act through oxidation and reduction reactions that form urea and free oxygen molecules, which cause erosion and increase enamel roughness. In addition, the intermediate products formed during these reactions can affect the interprismatic area by denaturing proteins in the organic enamel content [25]. Amorphous calcium and fluoride ions have been added to bleaching agents to reduce the mineral exchange in the enamel tissue during bleaching. Adding these molecules to bleaching agents has been reported to reduce mineral loss from the enamel [26].

Measuring phosphorus (P) and calcium (Ca) levels on the enamel surface provides valuable insight into the demineralization–remineralization dynamics. During bleaching, the dissolution of hydroxyapatite crystals leads to the loss of both P and Ca. Remineralization resumes when sufficient P and Ca are available in the saliva and the salivary pH remains neutral [27,28]. The ionic composition and replacement frequency of the artificial saliva were deemed likely to influence ion exchange and remineralization processes during the bleaching procedure. In this study, renewing the solution every 24 h ensured a stable ionic environment and prevented fluctuations in calcium and phosphate saturation levels. This stability contributed to controlled mineral deposition and dissolution dynamics. Similar findings have been reported in previous studies, emphasizing the role of saliva ionic strength (~0.08 mol/L) in maintaining mineral equilibrium and promoting enamel surface recovery following chemical challenges [29,30].

The cationic nature of chitosan plays a pivotal role in enhancing calcium ion (Ca^2+^) retention at the enamel interface through its electrostatic attraction to the negatively charged enamel surface. This electrostatic binding facilitates the formation of a protective mineral layer and promotes subsequent remineralization by increasing the local availability of calcium and phosphate ions [31]. These effects are consistent with earlier reports showing that chitosan-based systems strengthen enamel integrity and reduce mineral loss by reinforcing ionic interactions between bioactive compounds and hydroxyapatite [32].

When compared with other remineralizing agents, such as nanohydroxyapatite and casein phosphopeptide–amorphous calcium phosphate (CPP–ACP), the chitosan–theobromine combination demonstrates comparable or potentially superior performance in maintaining mineral stability following bleaching. Nanohydroxyapatite primarily acts by directly supplying calcium and phosphate ions to repair demineralized enamel regions [33], whereas CPP–ACP functions as a bioavailable calcium–phosphate reservoir that promotes remineralization by stabilizing casein phosphopeptide [34]. In contrast, chitosan’s cationic polymeric framework enhances ion adhesion and retention through electrostatic mechanisms, providing a synergistic advantage—particularly under post-bleaching conditions—in sustaining long-term enamel strength and resistance to demineralization [35].

In our study, the SEM-EDX evaluation performed after bleaching revealed that the Ca and Ca/P ratios decreased in the groups treated with FGM Whiteness Perfect (Group 4) and BioWhiten ProHome (Group 3). In contrast, the Ca and Ca/P ratios increased in the groups treated with the experimentally prepared agents containing 6% HP (Group 2) and 16% CP (Group 1) with theobromine and chitosan. P is another indicator of the demineralization–remineralization balance and was decreased in all groups in the intragroup comparison.

Soares et al. [36] reported decreased Ca and P concentrations in teeth treated with home bleaching agents containing 10% or 16% CP (FGM Whiteness Perfect). Consistently, in our study, Ca and P values decreased significantly after FGM Whiteness Perfect gel application.

Orilisi et al. [37] reported that BioWhiten bleaching gel containing 6% HP and nanohydroxyapatite applied for 50 min a day for 7 days resulted in a non-significant decrease in Ca and P levels, and the Ca/P ratio remained constant. Conversely, in our study, the Ca and P values decreased significantly with BioWhiten, potentially because we applied the agent for 14 days instead of 7 days.

Cakir et al. [38] used agents containing 10%, 20%, or 35% CP in an in vitro study on 60 extracted teeth, and the mineral content evaluation revealed that all three bleaching systems reduced the Ca levels. Consistently, in our study, the Ca value decreased after the FGM gel bleaching treatment.

Vilhena et al. [39] used an SEM-EDX device to evaluate enamel Ca and P mineral concentration changes after three bleaching applications with a 10% CP. They observed a non-significant increase in Ca and a decrease in P in all groups after bleaching. This result is similar to the Ca and P changes observed with the ECP gel in our study.

Botelho et al. [40] evaluated the effect of home and office bleaching applications on enamel Ca and P after using agents containing 10% and 20% CP and those containing 35% and 38% HP. No significant changes in post-treatment Ca concentration were detected for any of the four gels; however, the lowest Ca concentration was observed for the gel containing 38% HP at 14 days after the start of treatment. This finding is consistent with our between-group comparison, in which the agents containing HP caused the greatest decrease in Ca concentration.

Cavalli et al. [41] reported that bleaching agents containing HP alone could not prevent demineralization by reducing the Ca/P ratio, while agents containing F or Ca did prevent demineralization. These results are in line with the knowledge that Ca and F reduce enamel demineralization [42]. In our study, the increased Ca/P ratio in the experimental gel-treated groups is likely due to the addition of chitosan and the natural remineralizing agent theobromine.

Musanje et al. [43] reported that using artificial saliva promotes remineralization and increases Ca and P concentrations. In addition, keeping the teeth in artificial saliva may decrease microhardness values after bleaching applications, and Ca and P values may increase after ion exchange with saliva [44]. Consistent with this information, Ca_2_–Ca_3_ and P_2_–P_3_ values increased in all groups in our study.

### 2.4. Color Analysis

Color evaluations were conducted using the CIEDE 2000 system, which detected an effective and perceptible color change in all groups. A visible change occurred in all groups within the first 7 days. The intergroup evaluation revealed no significant differences between the groups before treatment, on day 7 of treatment, or at 15 and 28 days after the beginning of the treatment.

Pavesi-Pini et al. [45] added chitosan to bleaching gels containing 35% HP and evaluated the effects on tooth properties and bleaching efficiency. They reported that bleaching efficiency was similar in all groups; however, slight surface changes were observed in the SEM images in all groups. They observed that the chitosan-containing gels resulted in a smaller change in surface density. Thus, they inferred that chitosan-enriched HP gels could reduce the negative effects of bleaching on teeth without affecting the bleaching efficacy.

Li et al. [46] reported that adding 0.05–0.2% chitosan to bleaching gels increased the duration of free radical release and the color brightness value by preventing hydroxyl radical removal and HP decomposition. Sürmelioğlu et al. [10] compared two experimental bleaching gels (6% HP + titanium dioxide and 6% HP + titanium dioxide + chitosan) with a 35% HP-containing bleaching agent and evaluated the color changes after two bleaching treatments. The agent containing 35% HP showed superior results after the first and second sessions, and after 14 days, the color change in the 6% HP + titanium dioxide + chitosan gel group was comparable to that in the 35% HP group. Similarly, in our study, color measurements were taken 14 days after the completion of the bleaching treatment and 1 day after the bleaching applications. The color changes increased in the groups treated with chitosan-containing agents, nearing those observed in Group 4, which demonstrated the highest ΔE_002_ value at 1 day; 14 days after the bleaching applications, the color change values in the chitosan gel-treated groups were similar to or higher than those in Group 4. Chitosan increases the agents’ hydrophilicity, decreasing the contact angle and increasing the wettability [47]. Additionally, because of its mucoadhesive properties, chitosan stabilizes HP and increases its penetration into the tooth [48]. Thus, we hypothesize that chitosan enhanced the color change effect of the experimental gels compared with the commercial gels with the same CP or HP content.

Delfino et al. [49] applied three different bleaching agents (10%, 16% CP, and 6.5% HP); the color change observed with the 16% CP gel at 21 days was similar to that obtained with the 10% CP gel and significantly higher than that obtained with 6.5% HP. In addition, the color changes on the 21st day were superior to those on the 7th day and similar to those on the 14th day. Another study evaluating home bleaching applications for 14 days revealed that 20% CP application induced a greater color change than 7.5% HP; however, after 10 weeks, the color change results did not differ between the two treatments [50].

Almeida et al. [51] evaluated color changes with a home bleaching gel containing 10% CP applied for 3 and 1.5 h and one containing 6% HP applied for 1.5 h and 45 min. The most effective treatment was 10% HP for 3 h, while the smallest color change occurred with 6% HP for 45 min. Similarly, we found that gels containing 16% CP led to a greater color change than those containing 6% HP.

While theobromine and chitosan are widely used in medicine and dentistry, their combined use in tooth bleaching has not been previously reported. This study is the first on this subject.

Theobromine and chitosan are presented as an alternative to the synthetic polymers added to bleaching gels on the market. Theobromine has recently been described as an alternative to the highly remineralizing fluorine, which is avoided due to its toxicity. Chitosan can be used as a thickening agent and a carrier with bioadhesive, antioxidant, and antimicrobial effects.

A preliminary version of this manuscript was published as a preprint on Research Square [52].

Among the limitations of the present study is the absence of a true untreated control group, in which specimens would be stored in only artificial saliva without exposure to bleaching agents. Including such a control would have provided a more precise baseline for assessing mineral stability and distinguishing the effects of bleaching from those of natural remineralization. Although this aspect was not part of the original experimental design, future investigations are planned to incorporate an untreated artificial-saliva control group to improve the comparative assessment of mineral changes and strengthen the validity of the findings.

## 3. Conclusions

In this study, all bleaching applications were effective across all groups. After bleaching, the Ca concentration increased, though not significantly, in groups treated with the experimental gels containing chitosan and theobromine. In contrast, Ca significantly decreased in groups treated with the BioWhiten Prohome (Group 3) and FGM Whiteness Perfect (Group 4) gels. In addition, a non-significant decrease in the P concentration was observed in the groups treated with the experimental gels, whereas a significant decrease was found in the groups treated with the BioWhiten Prohome (Group 3) and FGM Whiteness Perfect (Group 4) gels. Thus, in terms of color and mineral changes, the best results were obtained in the group treated with the ECP gel (Group 1).

## 4. Materials and Methods

The Gaziantep University Ethics Committee approved this study (report number 2022/309).

### 4.1. Preparation of Samples

**Figure 5:**
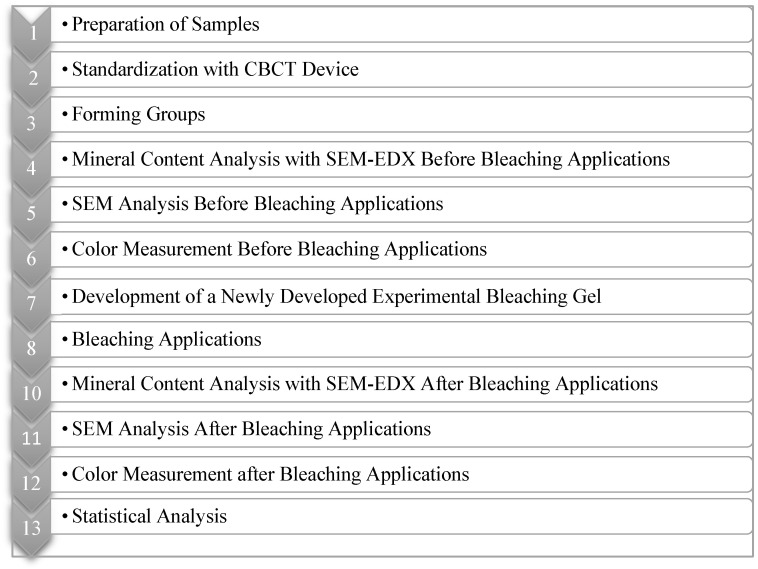
Flow Chart.

Before starting the study, a power analysis was performed to determine the number of samples required (G* Power 3.1.9.4). The minimum sample size was calculated as 48 for an effect size of 0.78 and a power of 95% (1 − β), with a 5% (α) confidence interval, for the mineral variation analysis. Likewise, for repeated color change measurements, the minimum sample size was 48 for an effect size of 0.81 and a power of 80% (1 − β) with a 5% (α) confidence interval. The study protocol is shown in Figure 5.

### 4.2. Selection of Teeth Used in the Study and Formation of Groups

Ninety-six central maxillary teeth were used in this study. All specimens were non-carious maxillary central incisors extracted for periodontal reasons. Only teeth with intact enamel surfaces and no visible cracks, restorations, or carious lesions were included, while any teeth exhibiting surface defects or discoloration were excluded. Immediately after extraction, the teeth were thoroughly cleaned of soft tissue debris and stored in 0.1% thymol solution at room temperature until use, in order to inhibit microbial growth and preserve enamel integrity. These inclusion criteria and storage procedures ensured sample uniformity and minimized variability in enamel morphology among groups.

**Figure 6:**
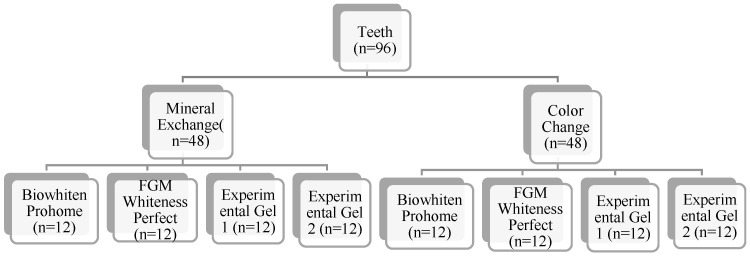
Groups.

All samples were standardized in terms of enamel–dentin thickness by CBCT (Promax 3D; Planmeca, Helsinki, Finland) and subsequently divided into 2 groups for the color determination (n = 48) and mineral change (n = 48) analyses. These 2 groups were then divided into 4 subgroups according to the whitening agent to be applied, as shown in Figure 6. No significant differences between the initial color measurements and mineral values were observed (n = 12).

### 4.3. Color Measurement with a Spectrophotometer Device

Color determination was performed with a VITA EasyShade Advance 4.0 (Zahnfabrik, Bad Sackingen, Germany) spectrophotometry device. Per the manufacturer’s instructions, measurements were made under D65 daylight on a gray background, with the device’s tip touching the center of the middle third of the tooth at the buccal surface. These measurements were repeated four times as follows: before the initiation of the bleaching treatment, on the 7th day of the treatment, 24 h after the end of the treatment, and 14 days after the end of the treatment. Holes with a diameter of 6 mm were drilled into 0.040-inch-thick (1.5 mm) hard plates to ensure standardization during color measurements. Each measurement was made three times through these holes, and the average of the obtained values was recorded. The color change was calculated using the CIEDE 2000 formula as follows: ΔE_00_ = [(ΔL/K_L_S_L_)^2^ + (ΔC/K_C_S_C_)^2^ + (ΔH/K_H_S_H_)^2^ + R.T(ΔC/K_C_S_C_)(ΔH/K_H_S_H_)]^1/2^ [23]. The parametric factors suggested by CIE (H_R_ = 1 K_C_ = 1 K_L_ = 1) were used [53]. Perceptible and acceptable color changes (50–50%) were taken as 0.8 and 1.8, with reference to the values used by Paravina et al. [53]

ΔL, ΔC, and ΔH in the formula represent lightness, chroma, and tonal differences, respectively. S_L_, S_C_, and S_H_ are weighting functions for the lightness, chroma, and tonal differences components, respectively. RT represents the interaction between hue and color differences in the blue region. K_L_, K_C_, and K_H_ are parameterized factors for changes in operating conditions [53].

### 4.4. SEM-EDX Analysis

An SEM-EDX (SEM, Zeiss Gemini SEM 300, Oberkochen, Germany) device was used in our study, which analyses the elemental nature of objects and existing surface defects using X-rays. Calcium (Ca) and phosphorus (P) levels were measured before the bleaching gel was applied, immediately after the bleaching procedure was completed, and 14 days after the treatment. In addition, 1 tooth was selected from each group, and the enamel surface was examined using the SEM (ZEISS Gemini SEM 300, Oberkochen, Germany) at ×600, ×1000, and ×2500 magnification. Mineral contents were examined by calibrating the device spectrum at 20 Kv, with ×100 magnification, a 5 nm spot size, a 35° departure angle, and a count of 100 s.

### 4.5. Preparation of the Experimental Bleaching Gel

Chitosan (2 g, Sigma Aldrich Chemical, St. Louis, MO, USA) was weighed on a precision balance (Sartorius, TE 214 S, Göttingen, Germany) and added to 100 mL water; 1% acetic acid was added to dissolve the chitosan with the help of a magnetic stirrer (FAITHFUL SH-2, Shanghai, China). Then, 0.05 g of theobromine (Sigma Aldrich Chemical, St. Louis, MO, USA) was added. The prepared solution was then mixed with 30% HP (Sigma Aldrich Chemical, St. Louis, MO, USA) at the determined ratios or a 6% HP gel and 97% CP (Sigma Aldrich Chemical, St. Louis, MO, USA) at the determined ratios to obtain a 16% CP gel.

### 4.6. Artificial Saliva Preparation

The prepared synthetic saliva solution contained 4380 mg sodium bicarbonate, 2540 mg potassium phosphate, 1640 mg potassium chloride, 882 mg calcium chloride, 9 mg sodium fluoride, 250 mg magnesium chloride, 20 mg nipagin, 200 mg nipazole, 48 mg sorbitol, 16 mg carboxymethylcellulose, and 2000 mL distilled water (pH 7.0). This artificial saliva solution was changed daily throughout the study. It had an ionic strength of approximately 0.08 mol/L, which was formulated to simulate the physicochemical environment of the oral cavity. The solution was replaced every 24 h to maintain chemical stability and minimize the accumulation of degradation products, and the pH (7.0 ± 0.1) was monitored daily. This replacement frequency was selected based on previous studies that demonstrated consistent ion balance and remineralization dynamics in similar experimental conditions [29,54].

### 4.7. Application of the Bleaching Procedure

**Figure 7:**
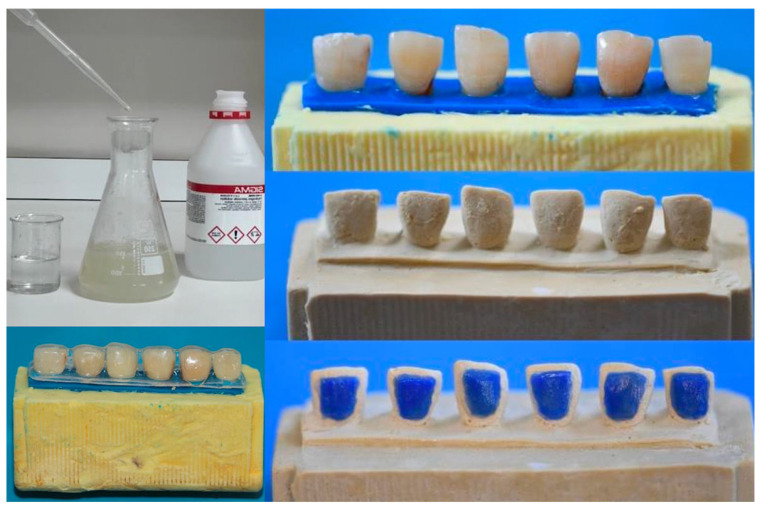
Preparation and application of bleaching agents.

The teeth were embedded in heavy-body silicone impression material (Optosil, Heraeus-Kulzer, Hanau, Germany) 2 mm above the enamel–cement boundary, with the crowns exposed. A border was created along the enamel–cement margin of the teeth with 1 mm light-body silicone impression material to prevent gel leakage and determine the gingival border (Optosil, Heraeus-Kulzer, Hanau, Germany). Then, the mold was measured using a suitable tray, and hard plaster was poured into alginate (Cavex CA37, Cavex Holland BV, RW Haarlem, The Netherlands). One-millimeter resin blocks (LC Block-Out Resin, Ultradent, South Jordan, UT, USA) were placed on the buccal surfaces of all teeth 1.5 mm from the determined gingival border to create a reservoir. The model was then polymerized with a wireless Valo Led (Ultradent Products, South Jordan, UT, USA) device, and a 0.035-inch (0.9 mm) bleaching plate was printed using a vacuum machine (Vacuum Forming Machine model no: 202, Keystone Industries, Myerstown, PA, USA). The borders of the plates were placed 1 mm below the anatomical enamel–cementum border of the teeth (at the determined gingival border). Bleaching gels were applied using this plate in a sterilization bag and a metal tub with a small amount of artificial saliva at the bottom; treatments were performed at 37 °C in an oven. Bleaching gels were applied without rinsing the artificial saliva from the teeth. After each bleaching application, all samples were washed with distilled water for 30 s, cleaned for 20 s with a soft toothbrush, and then kept in artificial saliva in an oven (Sanyo MOV-112S, Sanyo Electric Biomedical Co., Ltd., Osaka, Japan) at 37 °C for 14 days. This protocol is shown in Figure 7.

### 4.8. Statistical Analysis

The Shapiro–Wilk test was used to determine whether the numerical variables were normally distributed. ANOVA and post hoc least significant difference (LSD) tests were used to compare groups for normally distributed variables. A paired *t* test was used for in-group comparisons. Dunn and Kruskal–Wallis multiple comparison tests were used for the intergroup comparison of non-normally distributed variables, and the Wilcoxon signed-rank test was used for within-group comparisons. Analyses were performed using IBM SPSS 22.0 (IBM Corp., Armonk, New York, NY, USA), and *p* < 0.05 was considered to indicate significance.

## Figures and Tables

**Table 1:** Standard deviation and mean ΔE_00_ change values of all groups (ΔE ± sd).

	Group 1	Group 2	Group 3	Group 4
ΔE_001_	5.60 ± 2.27 ^Aa^	5.27 ± 2.49 ^Aa^	4.10 ± 1.51 ^Aa^	6.59 ± 2.31 ^Aa^
ΔE_002_	8.66 ± 4.22 ^Ba^	8.39 ± 4.37 ^Ba^	8.19 ± 4.16 ^Ba^	9.03 ± 4.63 ^Ba^
ΔE_003_	8.81 ± 3.35 ^Ba^	8.55 ± 3.49 ^Ba^	8.22 ± 3.62 ^Ba^	8.55 ± 3.70 ^Ba^

Letters in rows and columns indicate a statistically significant difference. Capital letters indicate statistical difference within the same column; lower case letters indicate statistical difference within the same row.

**Table 2:** Standard deviation and mean (%) values of Ca mineral for all groups (Ca ± sd).

	Group 1	Group 2	Group 3	Group 4
Ca_1_	33.85 ± 1.79 ^Aa^	34.17 ± 2.11 ^Aa^	32.07 ± 2.34 ^Aa^	31.86 ± 2.64 ^Aa^
Ca_2_	34.46 ± 1.54 ^Aa^	34.57 ± 1.90 ^Aa^	26.60 ± 3.55 ^Bb^	29.42 ± 3.95 ^Bb^
Ca_3_	34.57 ± 2.32 ^Aa^	34.77 ± 2.35 ^Aa^	28.89 ± 3.32 ^Bb^	29.61 ± 4.18 ^Bb^

Letters in rows and columns indicate a statistically significant difference. Capital letters indicate statistical difference within the same column, lower case letters indicate statistical difference within the same row.

**Table 3:** Standard deviation and mean (%) values of P mineral for all groups (P ± sd).

	Group 1	Group 2	Group 3	Group 4
P_1_	18.85 ± 1.14 ^Aa^	16.55 ± 1.42 ^Aa^	16.23 ± 2.17 ^Aa^	13.83 ± 1.45 ^Aa^
P_2_	18.37 ± 1.55 ^Aa^	16.21 ± 1.79 ^Aa^	15.48 ± 2.40 ^Ba^	12.47 ± 1.44 ^Ba^
P_3_	18.43 ± 1.82 ^Aa^	16.32 ± 1.63 ^Aa^	15.59 ± 1.97 ^Ba^	12.51 ± 1.35 ^Ba^

Letters in rows and columns indicate a statistically significant difference. Capital letters indicate statistical difference within the same column; lower case letters indicate statistical difference within the same row.

## Data Availability

We have all the survey data available.
